# Case Report: Yellow Fever Vaccine-Associated Neurotropic Disease and Associated MRI, EEG, and CSF Findings

**DOI:** 10.3389/fneur.2021.779014

**Published:** 2022-02-18

**Authors:** Michelle Cohen, Madeline Nguyen, Chad D. Nix, Brendan Case, Joshua P. Nickerson, Jacqueline Bernard, Julia Durrant, Delaram Safarpour, Tarvez Tucker, Kamila Vagnerova, William B. Messer

**Affiliations:** ^1^Department of Neurology, Oregon Health and Science University, Portland, OR, United States; ^2^School of Medicine, Oregon Health and Science University, Portland, OR, United States; ^3^Diagnostic Radiology, School of Medicine, Oregon Health and Science University, Portland, OR, United States; ^4^Department of Anesthesiology and Perioperative Medicine, Oregon Health and Science University, Portland, OR, United States; ^5^Department of Molecular Microbiology and Immunology, School of Medicine, Oregon Health and Science University, Portland, OR, United States; ^6^Program in Epidemiology, Oregon Health & Science University-Portland State University (OHSU-PSU) School of Public Health, Portland, OR, United States; ^7^Division of Infectious Diseases, Department of Medicine, Oregon Health and Science University, Portland, OR, United States

**Keywords:** yellow fever associated neurotropic disease, yellow fever vaccine, yellow fever virus, Creutzfeldt-Jakob Disease, vaccine adverse event, autoimmune encephalitis, post-infectious parkinsonism

## Abstract

Yellow fever vaccine-associated neurotropic disease (YEL-AND) is a rare and serious complication following vaccination with the 17D live attenuated yellow fever vaccine. Cases of YEL-AND have presented as acute inflammatory demyelinating polyneuropathy, acute disseminated encephalomyelitis, and meningoencephalitis. To date, intracranial imaging of the progression and resolution of this disease has been minimally depicted in the literature. We present the case of a 67-year-old woman who developed YEL-AND following vaccination. Her diagnosis was complicated by imaging findings consistent with variant Creutzfeldt Jakob Disease. Her clinical history and the progression of her intracranial imaging is discussed in this case report.

## Case Presentation

A 67-year-old woman with a history of atrial fibrillation and hyperlipidemia was transferred to our hospital for management of status epilepticus. She had no prior history of seizure, dementia or neuro-psychiatric disorder. Two months prior to presentation, she received CDC recommended vaccinations in anticipation of traveling to Guyana, South America, which included the 17D yellow fever virus (YFV) and Hepatitis B vaccines (1). Two weeks after vaccination she developed dizziness, double vision, sore throat and low-grade fever which she reported to her primary care provider (PCP). Five weeks later she presented to an outside hospital for evaluation of ongoing disequilibrium, vertical diplopia and numbness of her hands and lips. She was evaluated with a non-contrast computed tomography (CT) of the head which revealed age-related parenchymal atrophy without evidence of acute intracranial abnormality. The following morning, a magnetic resonance imaging (MRI) study of the head with and without gadolinium-based intravenous (IV) contrast, magnetic resonance angiography of the head and neck with and without contrast, and electroencephalography (EEG) demonstrated a few non-specific white matter changes, but were otherwise unremarkable. Comprehensive metabolic panel, complete blood count, erythrocyte sedimentation rate, C-reactive protein and random cortisol were all within normal limits. A lumbar puncture (LP) was performed and showed mildly elevated protein but no nucleated cells (Table 1); vitamin B12 levels were low in the 200 pg/mL range and repletion was begun. The patient was discharged home the next day. Three days later, her husband found her aphasic at home and she was taken back to the outside hospital. A repeat non-contrast CT of the head was again unremarkable without evidence of acute intracranial abnormality. Her labs were again normal, her aphasia improved and she was discharged home with concern for a functional neurologic disorder. She returned to the outside hospital within 48 h after the husband found her persistently aphasic and minimally interactive with right lower extremity shaking. She was admitted to the intensive care unit (ICU) with hypoxia and increased oral secretions requiring intubation for oxygenation and airway protection. This clinical picture, along with repeat EEG showing diffuse background slowing and “persistent left periodic lateralized epileptiform discharges suggestive of focal cortical excitability and an increased risk of seizures arising from that region,” prompted aggressive treatment with administration of levetiracetam and propofol infusion for tube tolerance as well as seizure control. At that time she was transferred to Oregon Health & Science University for continuous EEG monitoring as well as further workup and management.

**Table 1:** Patient CSF findings.

**Laboratory**	**LP 1 (1 day prior**	**LP 2**
**test**	**to admission)**	**(hospital day 2)**
White blood cells (cells/μL)	<1	7
Lymphocytes (%)	n/a	97
Protein (mg/dL)	54	43
Glucose (mg/dL)	51	66
Meningitis/encephalitis panel	Negative	Negative
Gram stain	Negative	Negative
Cryptococcal antigen	Not done	Negative
Cytology/Cytometry	Negative	Negative
14-3-3	Not done	Positive
T-tau protein	Not done	Positive
RT- QuIC	Not done	Negative
Paraneoplastic Autoantibodies	Not done	Negative
Encephalopathy Autoantibodies	Not done	Negative
**Cultures**
Bacterial	Negative	Negative
Fungal	Not done	Negative
Tick-Borne disease panel	Not done	Negative
Yellow Fever IgM	Not done	Not done
Serum IgG titers		1:640
CSF IgG titers		1:256

**Figure 1:**
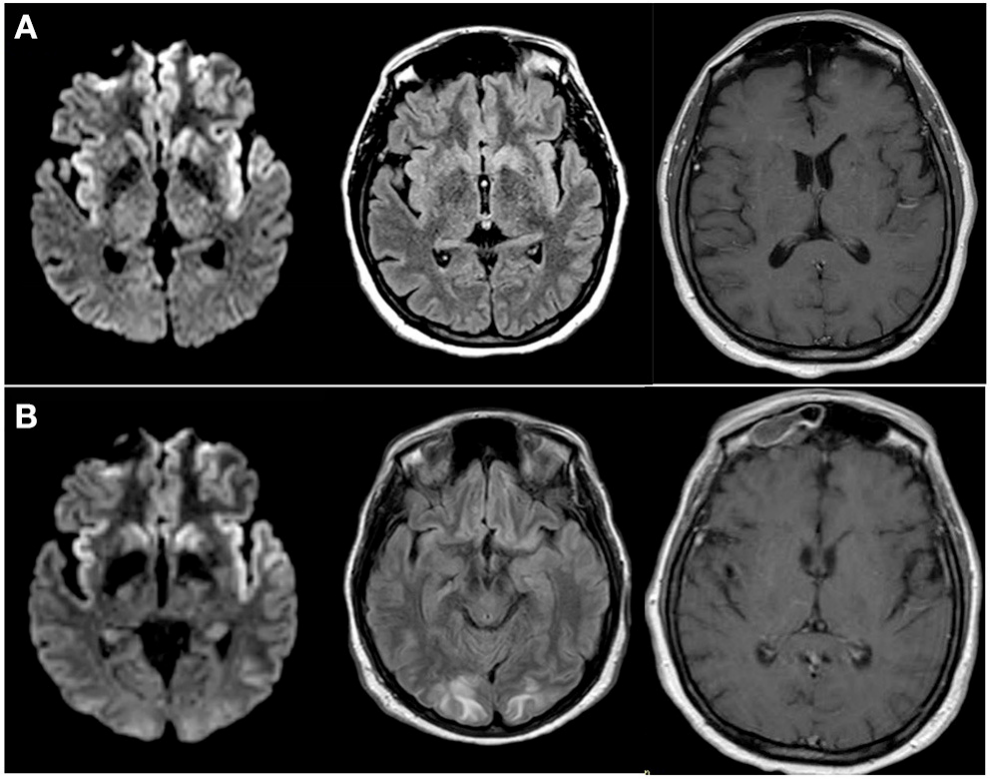
Progression of MRI Findings **(A)** MRI brain with and without intravenous contrast; Axial diffusion-weighted imaging (DWI) (left), FLAIR (middle) and T1 post-contrast (right) images obtained at the time of initial presentation to our institution demonstrated cortically-based increased signal within the paramedical frontal lobes and insular cortices bilaterally. Additional diffusion restriction is present within bilateral caudate heads and the left putamen. **(B)** Follow up MRI brain without contrast; axial DWI) (left), FLAIR (middle) and T1 post-contrast (right) images obtained 4 days later demonstrate unchanged cortically-based and basal ganglia diffusion restriction. FLAIR images also reveal new patchy subcortical white matter hyperintensity with involvement of the subcortical U-fibers. Contrast enhancement of pachymeninges seen diffusely.

**Figure 2:**
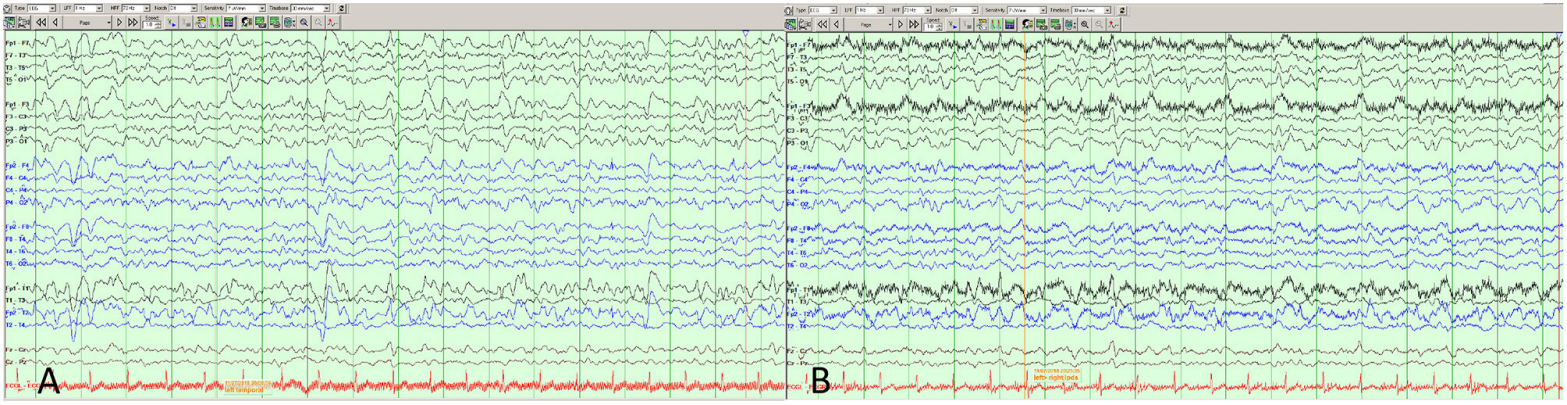
EEG in anterior to posterior bipolar montage showing **(A)** left temporal discharges with maximal electronegativity at T3 as well as **(A,B)** left greater than right ~1 hertz sharply-contoured lateralized discharges, frontally predominant, mostly biphasic with occasional triphasic morphology consistent with Laterialized Periodic discharges (LPDs, formerly called PLEDs) though at times they have more of a bihemispheric representation.

On admission to the Neurosciences ICU (NSICU), the patient was comatose. Her exam was notable for intact cranial nerve reflexes, witnessed rhythmic movement of the right lower extremity, triple flexion to noxious stimulus in both lower extremities, no movement of right upper extremity to noxious stimulus, hyper-reflexia throughout with bilaterally up-going toes and clonus at the right ankle. MRI with and without contrast (Figure 1A) demonstrated new diffusion restriction and mild T2/fluid-attenuated inversion recovery (FLAIR) hyperintensity along the left greater than right paramedian frontal lobes and insular cortices bilaterally as well as within the caudate heads and left lentiform nucleus. There was no associated enhancement or significant mass effect. While the predominantly gray matter signal abnormalities were suspicious for prion disease such as variant Creutzfeldt-Jakob Disease (vCJD), especially within the context of her EEG findings, given no preceding cognitive dysfunction, the imaging findings were favored to represent sequelae of recent epileptic activity. She received additional levetiracetam and placed on continuous EEG which showed rare epileptiform discharges in the left temporal region as well as bilateral lateralized periodic discharges (L > R) (Figure 2). Repeat LP was performed which appeared non-infectious with lymphocytic pleocytosis, with negative oligoclonal bands, cytology and cytometry. An infectious disease consult was obtained given recent YFV vaccination prior to presentation along with neurology consultation for seizure management and neuro-immunology given concern for potential inflammatory encephalitis. Additional cerebral spinal fluid (CSF) was sent to the Centers for Disease Control and Prevention (CDC) to evaluate for YFV antibodies as well as Mayo Clinic Laboratories to evaluate for autoimmune and paraneoplastic autoantibodies. The patient was started on empiric IV immunoglobulin (IVIG) at 0.4 mg/kg for 5 days while the remainder of workup was in process given the potential for YEL-AND (2).

A repeat MRI on day two of IVIG treatment (Figure 1B) revealed unchanged diffusion restriction and FLAIR signal abnormalities within the paramedian frontal lobes, insular cortices, and basal ganglia. At that time, however, a new patchy T2 and FLAIR hyperintense signal was seen in a symmetric distribution within the subcortical white matter of bilateral occipital lobes and precunei with involvement of the subcortical U-fibers without enhancement or mass effect. Diffuse pachymeningeal enhancement was also seen at that time, which was attributed to the patient's recent LP. Given the stability of the gray matter signal abnormalities, vCJD and sequelae of status epilepticus remained within the differential diagnosis. Additionally, the distribution of white matter involvement were considered potentially consistent with posterior reversible encephalopathy syndrome and progressive multifocal leukoencephalopathy. At this point, the decision was made to stop IVIG after three treatments given efficacy equivalence as well as increased risk with extra doses (possible posterior reversible encephalopathy syndrome-like picture on MRI, and transaminitis). A CT scan of her chest/abdomen/pelvis revealed no malignancy and paraneoplastic panels were negative.

The patient's neurological and respiratory exam slowly improved and she was extubated to high-flow nasal cannula for several days before safe to transition out of the NSICU to the acute care ward. Repeat MRI brain at time of floor transfer (hospital day 16) revealed near-complete interval resolution of the T2 and FLAIR signal abnormalities within the parietal and occipital lobe white matter, decreased diffusion restriction within the basal ganglia, but persistent diffusion restriction within the paramedian frontal and insular cortices. Pachymeningeal enhancement had also resolved, further supporting the prior LP as the etiology for those transient findings. Spot EEG at this time did not show any seizures though it continued to have moderate diffuse slowing as well as subtle focal slowing over the left hemisphere. Her NSICU course was otherwise complicated by *Escherichia coli* urinary tract infection which was treated with cefepime for 48 h and then narrowed to cephalexin to complete a 7-day course and *Streptococcus anginosus* bacteremia thought to be from a sinus infection based on CT sinus findings (10 day ampicillin/sulbactam course). Her seizures were well-controlled with levetiracetam 1,000 mg twice daily.

The remainder of her labs returned negative or within normal limits, including real-time quaking induced conversion, with the notable exception of: CSF protein 14-3-3 positive and elevated tau protein (>4,000); serum and CSF YFV IgM positive; YFV CSF 90% plaque reduction neutralization test (PRNT_90_) titer 1:256, and a serum PRNT_90_ titer of 1:640. CSF YFV polymerase chain reaction was not performed by the CDC given time elapsed post-vaccination. Given these results, it was felt that the positive 14-3-3 and tau were due to neuronal damage. The negative RT-QuIC ruled out vCJD. CSF YFV IgM positivity with titer levels close to those in serum confirmed a diagnosis of YEL-AND (3).

Neuro-immunology consultants recommended intravenous methylprednisolone at 1,000 mg per day for 5 days with a follow-up brain MRI after completion, which demonstrated continued improvement of the previously noted signal abnormalities with only mild persistent diffusion restriction and FLAIR hyperintensity within the frontal and insular cortices.

Her hospital recovery was complicated by swallowing apraxia and dysphagia requiring eventual percutaneous endoscopic gastrostomy tube placement, urinary retention requiring indwelling urinary catheterization, excess oral secretions treated with glycopyrrolate, and suspected frontal lobe apathy which improved mildly with low dose methylphenidate. At time of discharge to an inpatient rehabilitation facility, she was awake and oriented with choices, smiled appropriately to jokes but did not attempt spontaneous speech or movement and had dramatic bradyphrenia. She displayed a restricted affect and had positive palmo-mental reflex and grasp reflex in her right hand. She had a mild right lower facial droop, increased tone right-side greater than left with drift in the right upper extremity and reduced strength in all muscle groups on the right. Her reflexes were brisk but symmetric and she had now down-going toes bilaterally. She was able to walk with walker with stooped posture and significant bradykinesia. At discharge, the patient had a Montreal Cognitive Assessment (MoCA) score of 7/30.

The patient completed 4 weeks of intensive inpatient rehabilitation therapy and subsequently went to an adult foster home. She had moderately improved speech fluency and was able to ambulate with walker without support though she had continued significant psychomotor slowing. She was tolerating oral intake but was unable to meet caloric needs due to significant apraxia vs. apathy. At neurology clinic follow up visit 3 months after hospital discharge, MRI showed complete resolution of intracranial abnormalities previously seen. Due to the symptom constellation of bradyphrenia, bradykinesia, restricted affect and prior diffusion restriction in basal ganglia, diagnosis of post-infectious/post-inflammatory parkinsonism was made and she was started on levodopa-carbidopa. This was well-tolerated and increased as indicated over several months with improvement in all above symptoms. The patient has subsequently moved back home and is able to maintain caloric needs without tube feeding. Anti-epileptic therapy was successfully weaned without further seizure activity, frontal release signs (primitive reflexes) were absent on exam and repeat MoCA score were >20 on repeat clinic visits.

## Discussion

LP is rarely performed for symptomatic wild-type YFV disease as it is not classically associated with neurotropic illness (4). YEL-AND and other neurotropic manifestations following vaccination is most likely related to the manner in which 17D was attenuated to make the vaccine: serial passages through mouse and chick brains, leading to neuroadaptive but otherwise attenuating mutations (5, 6). YEL-AND remains a rare complication of 17D YFV vaccination with incidence estimated at eight individuals per one million vaccines administered (7, 8). Though a variety of encephalitic presentations for YEL-AND have been described including post-vaccinal encephalitis, aseptic meningitis, Guillain-Barre syndrome, meningoencephalitis, and acute disseminated encephalomyelitis, this is the first case where imaging, EEG and subacute rapidly progressive encephalopathy raised concern for prion disease, even though the time course was equivocal and no definitive exposure was established with comprehensive history. Additionally, reports of MRI imaging findings in YEL-AND have been limited to date. Similarly, our patient's EEG findings are notable when compared to other published cases which have either been unremarkable (*n* = 2), demonstrate disorganized background (*n* = 8), or revealed generalized low-amplitude slowing (4, 9). Recovery in most cases is complete in relatively quick fashion without significant sequelae (4) although deaths have been described in patients with YEL-AND (3), definitive attribution to the vaccine is difficult to establish (9). Our patient had a relatively prolonged course and while her imaging findings, seizures, and EEG did improve significantly relatively early in her hospital course, sequelae of intraparenchymal damage, specifically to the basal ganglia, complicated recovery with the development of Parkinsonism requiring dopaminergic therapy. Her clinical exam and functional status substantially lagged behind improvement in her imaging.

Comparison of this case to other case reports of YEL-AND (Table 2) (10) show patient factor similarities to the average and/or range in a variety of domains including time from vaccination to onset, serum WBCs, age, CSF WBC peak, and temperature, apart from a CSF lymphocytic count that was among the highest reported. We also suspect that her seizures and associated neuronal damage were more than a purely encephalitic process, possibly due to seizures and degree of neuronal damage. Additionally, while it is possible that her Parkinsonian symptoms were not due to her YEL-AND but instead presented as a true-but-unrelated syndrome, we think this is unlikely given resolution of her basal ganglia/putamen abnormalities at the time that she had clinical features of Parkinson's disease.

**Table 2:** Comparison of patient factors with known YEL-AND encephalitis average and ranges according to McMahon et al. report of 15 cases (10).

**Factor**	**Patient**	**YEL-AND Encephalitis**
		**Averages (10)**
Age (years)	67	54 (16–78)
Onset (days)	13	14 (5–2)
Temperature on admission (F)	99.0	101.9 (98.3–105)
WBC peak (cells/μL)	11.22	11.95 (6.3–15)
Creatinine peak (μmol/L)	0.77	1.4 (0.9–1.6)
WBC in CSF peak (cells/μL)	18	41.5 (0–406)
Lymphocytes in CSF peak (%)	97	27 (0–73)
Summary of CSF IgM (#positive/#tested)	1/1	5/6

## Conclusion

YEL-AND can present in a variety of ways and mimic a variety of other neurologic conditions, including prion disease. YEL-AND should be suspected in individuals with temporal relationship of symptom onset to vaccination, especially (but not exclusively) if patients are over the age of 65, with workup to include YFV IgM, sometimes IgG, and comparative serum to CSF titers to confirm the diagnosis. Clinical symptoms can persist beyond resolution of abnormalities in laboratory and clinical data including imaging.

## Data Availability Statement

The original contributions presented in the study are included in the article/supplementary material, further inquiries can be directed to the corresponding author/s.

## Ethics Statement

The study was reviewed by the OHSU Institutional Review Board and was granted a waiver of authorization (IRB# 00022903). Written informed consent was obtained from the individual(s) for the publication of any potentially identifiable images or data included in this article.

## Author Contributions

All authors listed have made a substantial, direct, and intellectual contribution to the work, and approved it for publication.

## Conflict of Interest

The authors declare that the research was conducted in the absence of any commercial or financial relationships that could be construed as a potential conflict of interest.

## Publisher's Note

All claims expressed in this article are solely those of the authors and do not necessarily represent those of their affiliated organizations, or those of the publisher, the editors and the reviewers. Any product that may be evaluated in this article, or claim that may be made by its manufacturer, is not guaranteed or endorsed by the publisher.
